# High‐resolution imaging for the detection and characterisation of circulating tumour cells from patients with oesophageal, hepatocellular, thyroid and ovarian cancers

**DOI:** 10.1002/ijc.29680

**Published:** 2015-08-19

**Authors:** Barry M. Dent, Laura F. Ogle, Rachel L. O'Donnell, Nicholas Hayes, Ujjal Malik, Nicola J. Curtin, Alan V. Boddy, E. Ruth Plummer, Richard J. Edmondson, Helen L. Reeves, Felicity E.B. May, David Jamieson

**Affiliations:** ^1^ Northern Institute for Cancer Research, Newcastle UniversityNewcastle upon TyneUnited Kingdom; ^2^ Newcastle upon Tyne Hospitals NHS Foundation TrustNorthern Oesophago‐Gastric Cancer UnitNewcastle upon TyneUnited Kingdom; ^3^ Queen Elizabeth HospitalNorthern Gynaecological Oncology CentreGatesheadUnited Kingdom; ^4^ Newcastle upon Tyne Hospitals NHS Foundation TrustNorthern Centre for Cancer CareNewcastle upon TyneUnited Kingdom; ^5^ Newcastle upon Tyne Hospitals NHS Foundation TrustThe Liver UnitNewcastle upon TyneUnited Kingdom; ^6^ Newcastle University Institute for AgeingNewcastle upon TyneUnited Kingdom

**Keywords:** circulating tumour cells, oesophageal adenocarcinoma, hepatocellular carcinoma, thyroid carcinoma, ovarian cancer, ImageStream^X^ imaging flow cytometry

## Abstract

Interest has increased in the potential role of circulating tumour cells in cancer management. Most cell‐based studies have been designed to determine the number of circulating tumour cells in a given volume of blood. Ability to understand the biology of the cancer cells would increase the clinical potential. The purpose of this study was to develop and validate a novel, widely applicable method for detection and characterisation of circulating tumour cells. Cells were imaged with an ImageStream^X^ imaging flow cytometer which allows detection of expression of multiple biomarkers on each cell and produces high‐resolution images. Depletion of haematopoietic cells was by red cell lysis, leukocyte common antigen CD45 depletion and differential centrifugation. Expression of epithelial cell adhesion molecule, cytokeratins, tumour‐type‐specific biomarkers and CD45 was detected by immunofluorescence. Nuclei were identified with DAPI or DRAQ5 and brightfield images of cells were collected. The method is notable for the dearth of cell damage, recoveries greater than 50%, speed and absence of reliance on the expression of a single biomarker by the tumour cells. The high‐quality images obtained ensure confidence in the specificity of the method. Validation of the methodology on samples from patients with oesophageal, hepatocellular, thyroid and ovarian cancers confirms its utility and specificity. Importantly, this adaptable method is applicable to all tumour types including those of nonepithelial origin. The ability to measure simultaneously the expression of multiple biomarkers will facilitate analysis of the cancer cell biology of individual circulating tumour cells.

## Abstract 2

**What's new?**

Circulating tumour cells (CTCs) are disseminated malignant cells from which biological and therapeutic information may be obtained non‐invasively. Detection of small CTC populations within the large number of normal blood cells is a challenge. This study describes a novel method for the detection and high‐resolution imaging of CTCs. Unlike most other studies, CTC detection is not reliant upon expression of a single biomarker. The method is applicable to all cancers; the authors present preliminary results from four tumour types. The high quality of the images allows biological characterisation of the tumour cells and increases the clinical potential of the approach.

Detection of circulating tumour cells (CTCs) was first reported more than a century ago.^1^ Interest in the clinical role of CTCs has increased with the development of improved technologies for their detection. The initial focus of research on cell‐based detection has been on the enumeration of CTCs. Studies have sought evidence that the burden of tumour cells in the circulation of patients with advanced cancer provides prognostic or predictive information. High numbers of CTCs in patients undergoing chemotherapy for metastatic breast, colorectal and prostate cancer are associated with poor patient prognosis.^2^, ^3^, ^4^

The introduction of novel agents that target‐specific molecular aberrations within cancer cells has driven exploration for biomarkers with which to inform accurate stratification of patients. Currently, biomarker profiles of tumour cells are measured on material obtained by surgical resection or invasive biopsy. CTCs are a source of disseminated malignant cells from which information about biological properties, or pharmacodynamic responses to novel therapeutics, may be obtained noninvasively.

Effective enumeration and characterisation depend upon a reliable method for the evaluation of CTCs. Detection of small populations of CTCs within the large number of normal blood cells represents a significant technical challenge. A single CTC may be detected in 7.5 ml of blood,^5^ a volume that may contain up to 75 million leukocytes and 50 billion erythrocytes. Enrichment of the sample is required before tumour cell identification and characterisation can occur. This enrichment may consist of positive selection of the CTCs, positive depletion of the normal blood cells or a combination of the two approaches. A perfect method would remove completely the red blood cells, white blood cells and platelets, produce no cellular debris and recover all the CTCs. The majority of clinical studies reported have relied upon positive selection of tumour cells that express a single biomarker.^6^, ^7^, ^8^, ^9^, ^10^, ^11^, ^12^, ^13^ The antigen chosen most commonly is epithelial cell adhesion molecule (EpCAM).

We sought to develop a method for identification, quantification and characterisation of CTCs that would not rely upon expression of a single antigen and would be applicable to multiple tumour types. Additional aims were to obtain high‐resolution images of the cells, avoid cell damage, achieve high sensitivity and specificity and have the ability to analyse the expression of multiple biomarkers in each tumour cell. We report the development of our method, and its validation with whole blood from patients with oesophageal, hepatocellular, thyroid and ovarian cancers.

## Materials and Methods

### Tissue culture

SK‐GT‐4 oesophageal adenocarcinoma cells (DSMZ, Braunschweig, Germany) and OVCAR‐3 ovarian cancer cells (ATCC) were grown in RPMI supplemented with 10% foetal calf serum (FCS). ML1 thyroid cells (DSMZ, Braunschweig, Germany) were grown in DMEM supplemented with 10% FCS (Life Technologies, Paisley, UK). Huh‐7 hepatocellular carcinoma cells (ATCC) were grown in DMEM with F12 Ham's nutrient and 10% FCS. All other reagents were purchased from Sigma‐Aldrich (Poole, UK) unless stated otherwise.

Cells were maintained in exponential growth at 37°C in a humidified atmosphere, supplied with 5% CO_2_ and discarded after the 30th passage. Cells were confirmed to be mycoplasma free (MycoAlert mycoplasma detection kit; Lonza).

### Immunofluorescence

Cells were fixed by incubation in 0.4% formaldehyde for 20 min, or Phosflow Lyse/Fix buffer (BD, Oxford, UK) and permeabilised by incubation in Perm/Wash buffer (BD, Oxford, UK) for 1 hr at room temperature. Cells were incubated with 1:20 antipan‐cytokeratin (clone C‐11) PE (Cayman Chemical) and appropriate tumour‐specific intracellular antibodies for 30 min at room temperature: 1:50 antisurvivin Alexa Fluor^®^ 647 (Cell Signaling); 1:50 antiMUC16 which is known as carcinoma antigen 125 (CA‐125) (Abcam) Alexa Fluor^®^ 594, conjugated with an APEX™ kit (Invitrogen) as per manufacturer's instructions; 1:50 antialpha faetoprotein Alexa Fluor^®^ 594 (Cell Signaling). Membrane antibodies and nuclear stains were added and incubated for 1 hr at room temperature: 1:20 anti‐CD45 (clone H130) V450 or PE:Cy7 (BD Biosciences); 1:20 anti‐EpCAM CD326 (clone 9C4) Alexa Fluor^®^ 488 (Biolegend); DAPI or DRAQ5 (Biostatus, UK). Cells from thyroid cancer patients were incubated first with 1:50 antisodium:iodide symporter (NIS; Milipore) followed by anti‐mouse Texas Red (Life Technologies, UK). Subsequent incubations were as described above except that the anti EpCAM CD326 (clone 9C4) was conjugated to PerCP:Cy5.5 (Biolegend). Cells were washed in 500 µl of Perm/Wash buffer and recovered by centrifugation at 500 g for 5 min and either analysed immediately or stored as a pellet at 4°C until analysis.

### High‐resolution flow cytometry

Cells were resuspended in phosphate‐buffered saline (PBS) and divided into 60 µl aliquots. Aliquots were analysed with an ImageStream^X^ (Amnis) image flow cytometer with an 8‐µm core at 60 mm/sec with 7% Speed Beads^®^. Speed Beads^®^ are a 1 μm polystyrene beads that allow calibration of the flow and focus of the ImageStream^X^. Fluorochromes were excited with 405, 488, 561 and 642 nm lasers and light emitted by the fluorescently‐labelled cells was collected through a ×40 objective. Of the twelve channels available, channels 1 and 9 were reserved for brightfield images. The other channels were set to collect magnified emitted light with two CCD cameras, each spatially resolved into five distinct spectral bandwidths, over a range of wavelengths between 430 and 745 nm. Single‐colour reference samples for each fluorochrome were generated by inclusion of cells that had been incubated with each antibody separately. A compensation matrix was built with the data from single‐colour reference samples to allow removal of spectral overlap to adjacent channels from each detection channel. The diameters of individual cells detected were measured and are given as means ± standard errors of the mean for different populations of cells.

### Detection of cells in whole blood

Ethical approval for the study was obtained from the Newcastle and North Tyneside Research Ethics Committee. An initial predraw of 4 ml of blood was discarded to reduce contamination with epithelial skin cells. Blood samples were collected in Transfix collection tubes (Cytomark, UK) to store for up to 24 hr at 4°C or BD Vacutainer EDTA tubes (BD Biosciences) for immediate use.

For each cell recovery experiment, 12 ml‐blood samples were collected from healthy volunteers. Cells from cancer cell lines were trypsinised and resuspended in media. Cells were counted with an improved Neubauer haemocytometer (Hawksley, UK) and were diluted twice by 1 in 10. The appropriate volume of cells containing 2,000, 200 or 20 cells was added to 4 ml blood samples to give 500, 50 and 5 malignant cells/ml of whole blood.

To determine the contribution of the final stage of the imaging flow cytometric analysis to the overall recovery rates, 500 unprocessed cells were analysed directly with the ImageStream^X^ flow cytometer. Cells were imaged in the brightfield channel and images with cellular morphology were counted.

### Analysis of patient samples

Whole blood samples were obtained from patients undergoing treatment for oesophageal adenocarcinoma, hepatocellular carcinoma, thyroid carcinoma or ovarian cancer at the Newcastle‐upon‐Tyne and Gateshead NHS Foundation Trusts, UK. Patient clinical data was recorded for each patient in accordance with ethical approval. In addition whole blood samples were obtained from healthy volunteers who had no medical history of any current or previous cancer. CTCs were defined by the presence of at least one tumour‐specific antigen, the absence of CD45, the presence of a nucleus and cellular morphology as assessed by brightfield imaging. CTC images were verified independently by two members of the research group.

### Depletion of haematopoietic cells

Patient and healthy volunteer samples were processed to enrich for nonhaematopoietic cells prior to analysis. Cells were transferred into 50 ml Falcon tubes, incubated in 5 ml of 5% bovine serum albumin (BSA) in AutoMACS rinse solution (Miltentyi Biotec, Germany). Human FcR blocking reagent (Miltenyi Biotec, Germany) was added directly to the blood to a final dilution of 1:40 to prevent nonspecific antibody binding. Red blood cells were lysed, and all other cells fixed, by incubation in BD Phosflow Lyse/Fix buffer 1:20 (v:v; BD Biosciences) for 15 min at 37°C. Fixed, unlysed cells were collected by centrifugation at 500 g at room temperature for 8 min. The supernatant was discarded and the cells were resuspended in 500 µl RoboSep buffer (Stemcell Technologies, UK) in a polystyrene Falcon tube (BD Biosciences).

White blood cells were removed with an EasySep human CD45 depletion kit (Stemcell Technologies, UK) as per the manufacturer's instructions. Briefly, antibodies against CD45, bound in tetrameric complexes were added to the cell suspension and incubated for 15 min at room temperature. Dextran‐coated magnetic nanoparticles were added and incubated with the cells for 10 min at room temperature. The cell suspension was diluted in 5 ml of Robosep buffer and placed in an EasySep™ big easy magnet (Stemcell Technologies, UK) for 10 min at room temperature. The unretained cell fraction was decanted into a clean tube by inversion of the sample and magnet. The recovered cells were centrifuged at 250 g for 5 min, resuspended in 1 ml Perm/Wash buffer (BD, Oxford, UK), incubated for 1 hr at room temperature and processed for analysis by image flow cytometry.

## Results

### Image flow cytometry of malignant cells

Four distinct tumour types were selected for development of a universal method for detection of CTCs. Expression of EpCAM and cytokeratins 4, 5, 6, 8, 10, 13 and 18 was chosen for detection of the malignant cells. In addition, detection of expression of tumour‐ or tissue‐specific markers was included: for hepatocellular carcinoma, alpha‐faetoprotein; for thyroid carcinoma, thyroglobulin and sodium:iodide symporter (NIS) and for ovarian cancer, cancer antigen 125 (CA‐125). There is no accepted tumour‐specific marker for oesophageal adenocarcinoma, but survivin expression is reported to be high and measurement of its expression was included for this tumour type.

**Figure 1:**
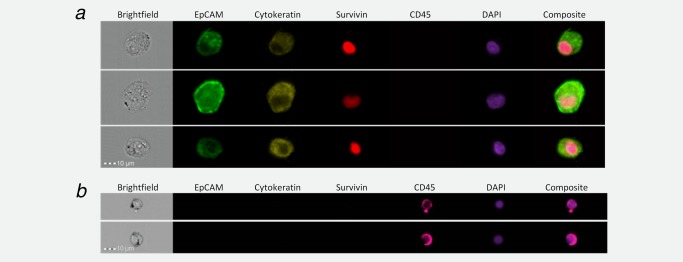
EpCAM, cytokeratin, survivin and CD45 expression in oesophageal adenocarcinoma cells and in white blood cells. SK‐GT‐4 cells were grown to 80% confluence in routine culture medium, trypsinised and 1 × 10^6^ cells fixed with 1% formalin. Cells were permeabilised by incubation with 0.3% saponin, incubated overnight with antibodies against cytokeratins 4, 5, 6, 8, 10, 13 and 18, and CD45, and incubated subsequently with antibodies against EpCAM and survivin. Cells were washed, re‐suspended in 100 μl and 2 μl DAPI added. Cells were visualised with an ImageStream^X^ flow cytometer with the lasers set to emit excitation at 405, 488, 561 and 658 nm (*a*). Red blood cells were removed from whole blood by ammonium chloride lysis, the remaining blood cells were concentrated by centrifugation, fixed, permeabilised and incubated with antibodies against EpCAM, cytokeratins 4, 5, 6, 8, 10, 13 and 18, survivin and CD45 as in (*a*). Blood cells were concentrated, incubated with DAPI and visualised (*b*). Images were collected with a ×40 objective with the wavelengths for the collection channels set at: 480–560 nm, EpCAM; 560–595 nm, cytokeratins; 745–800 nm, CD45; 430–505 nm, DAPI; and 642–745 nm, survivin.

Initially, detection was optimised with established cell lines. SK‐GT‐4 oesophageal adenocarcinoma cells were incubated with fluorescently‐conjugated antibodies against EpCAM, cytokeratins 4, 5, 6, 8, 10, 13 and 18, and with DAPI, a fluorescent dye that binds DNA. A fourth conjugated antibody against leukocyte common antigen CD45 was included because it would allow subsequent discrimination of leukocytes. Cells were analysed for expression of the antigens with an ImageStream^X^ image flow cytometer. Representative images from three cells are shown in Figure 1 *a*. The different images obtained from a single cell are shown in each horizontal panel. The brightfield image of the cell shown on the left hand side of each panel allows visualisation of the nucleus, plasma membrane and overall cell morphology. All three cells have cytoplasmic immunoreaction for EpCAM, with some evidence of intracellular vesicular accumulation and membrane localisation. The immunoreaction for the cytokeratins was less intense, but evident in the cytoplasmic compartment of all cells. Survivin expression was detected in the nuclei of the cells coincident with the localisation of the DAPI DNA dye. The composite images shown on the right hand side of the panels confirm localisation of EpCAM and the cytokeratins in the cytoplasmic and membrane compartments of the cells and the distinct localisation of survivin in the nuclei. Expression of CD45 was not detected in SK‐GT‐4 oesophageal adenocarcinoma cells.

Hepatocellular Huh‐7 cells, ML1 thyroid cells and OVCAR 3 ovarian cells were analysed as described above except that the antibody against survivin was replaced with antibodies against alpha‐faetoprotein, thyroglobulin and NIS, and CA‐125, respectively. Images of comparable quality to those shown in Figure 1 *a* were obtained for all three cell lines (data not shown). Alpha‐faetoprotein, thyroglobulin and NIS, and CA‐125 were detected in Huh‐7, ML1 and OVCAR 3 cells, respectively. These results demonstrate the applicability of the method to the detection of multiple tumour types, the measurement of tumour‐type‐specific biomarkers and the high quality of the images that may be obtained.

### Detection of malignant cells in, and recovery from, whole blood

It was important to demonstrate the specificity of our method with whole blood from healthy individuals. Blood was collected, red blood cells were lysed and the remaining blood cells collected by centrifugation. These blood cells were incubated with antibodies against EpCAM, cytokeratins 4, 5, 6, 8, 10, 13 and 18, survivin and CD45, centrifuged at low g‐force to remove platelets and analysed for expression of the antigens by image flow cytometry (Fig. 1 *b*). The brightfield images demonstrate that the cells detected are of smaller diameter (12.2 ± 0.2 µm) than the SK‐GT‐4 oesophageal cells (20.3± 0.2 µm; unpaired *t* test, *p* < 0.001); nearly all express CD45. Fifty‐three blood samples from healthy individuals have been analysed with the tumour‐specific antibodies. No cells were detected with morphology consistent with a malignant cell and expression of a tumour‐specific antigen. These results indicate that the detection method will discriminate effectively nonhaematopoietic cells from haematopoietic cells.

Despite the ability of the ImageStream^X^ flow cytometer to image 2,000 cells/sec, the large number of cells in whole blood means that analysis is extremely lengthy and produces an enormous amount of data for analysis and storage. We investigated the best method with which to enrich blood for nonhaematopoietic cells. For these experiments, known numbers of cultured malignant cells were added to samples of whole blood and then the malignant cells were purified and analysed by image flow cytometry. Positive selection of the malignant cells involved identification of a universally expressed antigen or differential separation on the basis of density. All methods tested gave low recovery of the malignant cells and the cells that were recovered were damaged physically as assessed by the images produced (data not shown). We therefore developed a method for positive depletion of haematopoietic cells. After initial lysis of the erythrocytes; the leukocytes, platelets and malignant cells were incubated with tetrameric antibody complexes against CD45 and dextran‐coated magnetic particles. The cells were placed in a magnet and those not attracted to the magnet were recovered. The method was optimised to minimise loss of nonhaematopoietic cells whilst maximising depletion of CD45 positive cells.

**Figure 2:**
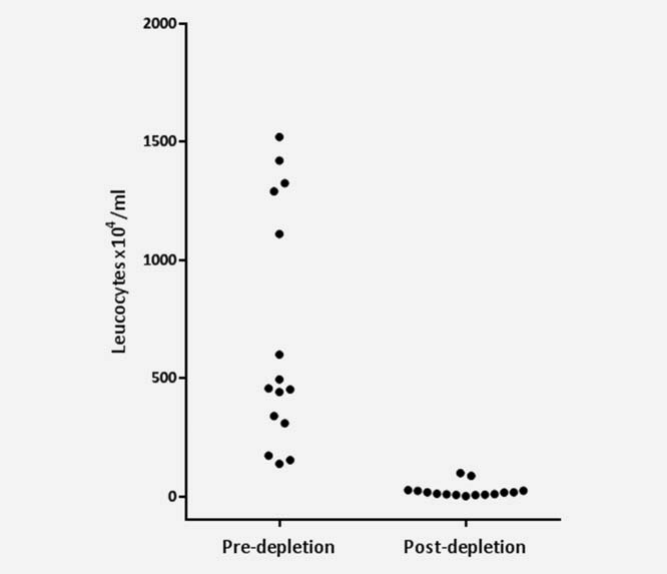
Enrichment for nonhaematopoietic cells. Cultured malignant cells were added to 4 ml of whole blood and cells were incubated with tetrameric antibody complexes against CD45 and dextran‐coated magnetic particles for 1 hr and placed in an EasySep Big Easy magnet. Cells not attracted to the magnet were recovered. The number of leukocytes was counted with a haemocytometer prior to and after depletion. Erythrocytes were removed by lysis and platelets by centrifugation at 250 g.

Depletion of leukocytes was consistently 95 ± 0.8% (Fig. 2). The recovered cells were incubated with detection antibodies and DAPI, centrifuged at 250 g to remove platelets and analysed by high‐resolution image flow cytometry as described above. Analysis of cells enriched from 1 ml of blood takes 20 min in the ImageStream^X^ flow cytometer whereas analysis of 1 ml of blood without prior enrichment takes 180 min. Images of the malignant cells and residual leukocytes were similar to those shown in Figure 1, which confirms that minimal damage to the malignant cells is caused by this enrichment method of positive blood cell depletion (data not shown).

**Figure 3:**
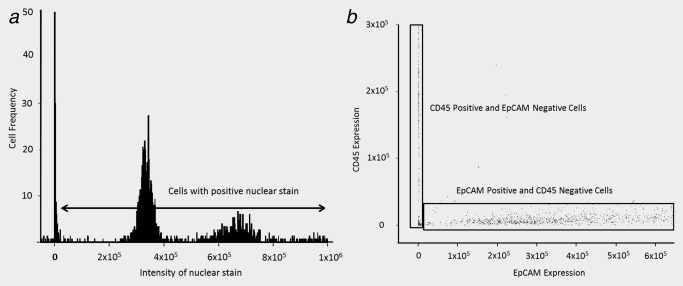
Discrimination of the malignant cell population in whole blood from the residual leukocytes after positive depletion of blood cells. Cultured malignant SK‐GT‐4 cells were added to 4 ml of whole blood. Erythrocytes were removed by ammonium chloride lysis and platelets by centrifugation at 250 g. The cells in the supernatant were incubated with tetrameric antibody complexes against CD45 and dextran‐coated magnetic particles for 1 hr and placed in an EasySep Big Easy magnet. The cells not attracted to the magnet were recovered and analysed in an ImageStream^X^ flow cytometer as described in the legend to Figure 1. The number of images at each DAPI fluorescence intensity is shown (*a*). The intensity of fluorescence for the CD45 antibody is compared with the intensity of fluorescence of the EpCAM antibody for all cells distinguished (*b*).

The malignant cells are distinguished from residual leukocytes by expression of epithelial cell‐ and tumour‐specific antigens, absence of expression of CD45, and by their morphology and larger size. Analysis of the images obtained with the IDEAS Software enables automatic discrimination of the two cell populations. The first selection is based upon the intensity of the nuclear dye retained by the cells (Fig. 3 *a*). The sharp peak around zero contains beads and small particles of debris. The peak of intensity between 3 and 4 × 10^5^ fluorescence units contains single leukocytes. The third peak of intensity between 5 and 8 × 10^5^ fluorescence units contains malignant cells and doublets of white blood cells and is analysed further. Subsequent selection is based upon absence of CD45 expression and presence of expression of EpCAM, cytokeratins and survivin, alpha‐faetoprotein, thyroglobulin, NIS or CA‐125. The effectiveness of the discrimination is illustrated in Figure 3 *b*; one population of cells expresses CD45 but not EpCAM while the second population expresses EpCAM but not CD45. Cells that express one or more of the epithelial‐ or tumour‐specific antigens and do not express CD45 are selected automatically with the IDEAS Software. The images of each of these cells are examined visually to confirm that they have a cellular morphology as shown in Figure 1 and that the IDEAS Software is able to distinguish the malignant cells from any residual haematopoietic cells.

We processed, imaged and analysed 5 ml blood samples from 24 healthy volunteers as described above. Blood was enriched by CD45‐positive cell depletion, incubated with the detection antibodies and analysed by ImageStream^X^ flow cytometry. The majority of the residual white blood cells were detected with the CD45 antibody. None of the cells imaged within the healthy volunteer samples met the criteria for classification as a CTC.

**Figure 4:**
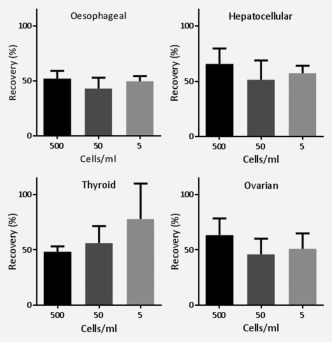
Retrieval of malignant cells from whole blood. SK‐GT‐4, Huh‐7, ML1 and OVCAR 3 cells were added to 4 ml of whole blood to give a final concentration of 500, 50 and 5 malignant cells/ml. The samples were enriched for malignant cells by depletion of blood cells and the residual cells were incubated with fluorescent antibodies and nuclear dye and analysed by high‐resolution flow cytometry as described in the Materials and Method. The mean recoveries (±SEM) across the four tumour types were 57.3 ± 3.6, 49.2 ± 3.9 and 59.0 ± 5.6% from 500, 50 and 5 cells/ml of blood, respectively. Experiments were replicated at least thrice.

The efficiency of recovery of malignant oesophageal, hepatocellular, thyroid and ovarian cells from whole blood was evaluated. The cell recovery across the four tumour types was 57.3 ± 3.6, 49.2 ± 3.9 and 59.0 ± 5.6% from 500, 50 and 5 cells/ml of blood, respectively (Fig. 4). We investigated specifically the recovery during the final analysis with the image flow cytometer. The recovery during analysis of known numbers of cells by image flow cytometry was 89.2+/−6.2%, which means that the recovery during the red blood cell lysis, white blood cell depletion, centrifugation and antibody labelling steps was 61.9% to give an overall recovery of 55.2%.

### Detection and analysis of circulating tumour cells

**Figure 5:**
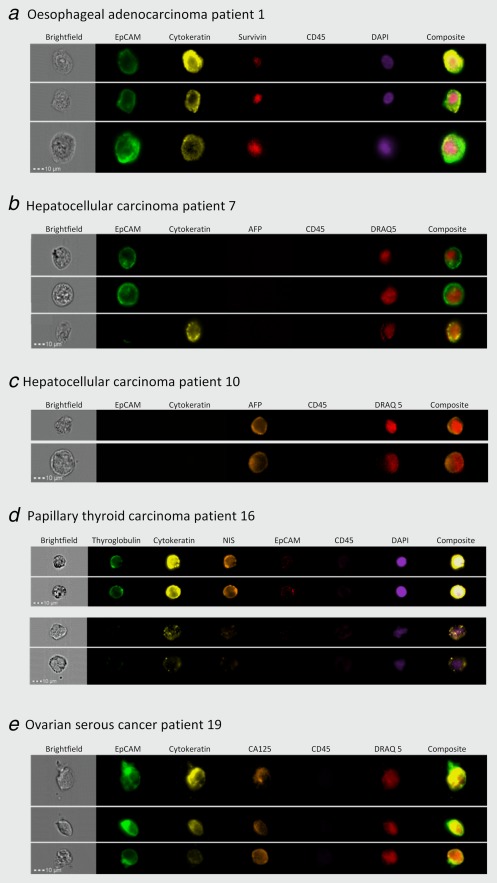
Detection of circulating tumour cells in blood from patients with oesophageal, hepatocellular, thyroid and ovarian cancers. Blood was obtained from patients, enriched for nonhaematopoietic cells and incubated with antibodies against EpCAM, cytokeratins 4, 5, 6, 8, 10, 13 and 18, CD45 and either survivin (oesophageal; *a*), alpha‐faetoprotein (AFP) (hepatocellular; *b* and *c*), thyroglobulin and sodium:iodide symporter (thyroid; *d*) or CA‐125 (ovarian; *e*). Cells were incubated with DAPI (*a* and *d*) or DRAQ5 (*b*, *c* and *e*) and analysed by high‐resolution flow cytometry. Representative images of cells are shown. Small particles visible in some brightfield images represent the Speed Beads^®^ that are added to allow calibration of the objective and camera.

To validate the method, blood samples from six individual patients with oesophageal, hepatocellular, thyroid and ovarian cancer were analysed essentially as described above. Representative images of tumour cells detected for each of the four tumour types are shown in Figure 5. The morphology of the oesophageal tumour cells detected was similar to that of the cultured cells. All of the oesophageal CTCs detected expressed EpCAM, cytokeratins and survivin. CTCs were detected in two of the six oesophageal adenocarcinoma patients analysed (Table 1). None of these patients had macroscopic evidence of metastatic disease. The mean diameter of all the circulating oesophageal adenocarcinoma cells detected was 17.2 ± 0.4 µm.

**Table 1:** Patient demographics and numbers of circulating tumour cells in blood from patients with oesophageal, hepatocellular, thyroid and ovarian cancers

Patient no.	Tumour origin	Tumour type	Sex	Age	Disease stage^a^	Distant metastatic disease	Previous therapy	CTCs detected^b^	CTCs in 7.5 ml
1	Oesophagus	Adenocarcinoma	Male	59	T2N2M0	No	No	43	64
2	Oesophagus	Adenocarcinoma	Male	63	T3N2M0	No	No	17	27
3	Oesophagus	Adenocarcinoma	Male	83	T1N0M0	No	No	0	0
4	Oesophagus	Adenocarcinoma	Female	74	T3N1M0	No	No	0	0
5	Oesophagus	Adenocarcinoma	Male	65	T2N0M0	No	No	0	0
6	Oesophagus	Adenocarcinoma	Male	72	T3N2M0	No	No	0	0
7	Liver	Hepatocellular carcinoma	Male	70	T1N0M0	No	TACE^c^/SIRT^d^	20	37
8	Liver	Hepatocellular carcinoma	Female	62	T2N0M0	No	TACE^c^	0	0
9	Liver	Hepatocellular carcinoma	Male	93	Unknown	No	No	0	0
10	Liver	Hepatocellular carcinoma	Male	75	T3bN0M0	No	No	9	16
11	Liver	Hepatocellular carcinoma	Male	81	T1N0M0	No	TACE^c^	2	4
12	Liver	Hepatocellular carcinoma	Female	85	T1N0M0	No	TACE^c^	2	4
13	Thyroid	Medullary thyroid cancer	Male	49	T2N2M1	Yes	Surgery	4	4
14	Thyroid	Multifocal follicular variant of papillary thyroid cancer	Female	29	T1aN0M0	No	Surgery Thyroxine	0	0
15	Thyroid	Follicular thyroid cancer	Female	47	T2N0M0	No	Surgery Thyroxine Radioiodine	1	1
16	Thyroid	Papillary thyroid cancer	Male	61	T4N2M1	Yes	Surgery Thyroxine Radioiodine radiotherapy	118	118
17	Thyroid	Follicular variant of papillary thyroid cancer	Female	52	T3N0M0	No	Surgery Thyroxine Radioiodine	0	0
18	Thyroid	Follicular variant of papillary thyroid cancer	Female	50	T2N0M0	No	Surgery Thyroxine Radioiodine	1	1
19	Ovary	Serous	Female	50	FIGO Stage 4	Yes	No	30	45
20	Ovary	Serous	Female	66	FIGO Stage 3c	Yes	No	15	23
21	Ovary	Mucinous	Female	75	FIGO Stage 3c	Yes	No	5	8
22	Ovary	Serous	Female	54	FIGO Stage 3c	Yes	No	0	0
23	Ovary	Serous	Female	85	FIGO Stage 4	Yes	No	4	6
24	Ovary	Serous	Female	72	FIGO Stage 3c	Yes	No	0	0

a Disease stage is provided according to the latest UICC TNM classification for oesophageal, hepatocellular and thyroid cancer, and the FIGO classification is shown for ovarian cancer.

b The volume of blood analysed was 5 ml for patients with oesophageal and ovarian cancers, 4 ml for those with hepatocellular carcinoma and 7.5 ml for thyroid cancer patients.

c Transarterial chemoembolisation.

d Selective internal radiotherapy treatment.

CTCs were detected in blood from four out of six patients with hepatocellular carcinoma (Figs. 5 *b* and 5 *c*). Some of the CTCs from patient seven expressed EpCAM but cytokeratins were not detected, and other cells expressed cytokeratins but EpCAM expression was not detected. None of the CTCs from this patient expressed alpha‐faetoprotein. Alpha‐faetoprotein was detected in cells from patient ten that did not express EpCAM or the cytokeratins (Fig. 5 *c*). The morphology of the hepatocellular tumour cells varied. The diameter of the hepatocellular carcinoma cells was 21 ± 0.6 µm which is significantly larger than the diameter of the CTCs from all three other tumour types (unpaired *t* test, *p* < 0.001)

CTCs were detected in three of the six patients with thyroid cancer. The majority of these tumour cells expressed cytokeratins, thyroglobulin and NIS. EpCAM expression was low or undetectable. The highest number of CTCs was detected in blood from a patient with known metastatic disease. A third of their CTCs had clear membrane and cytoplasmic immunoreactivity for thryoglobulin, NIS and cytokeratins, no obvious morphological damage and well‐defined oval nuclei (Fig. 5 *d*). These CTCs stained intensely with DAPI possibly because they were aneuploid or were in the G2 stage of the cell cycle. The other cells expressed lower levels of cytokeratins, did not express detectable levels of thyroglobulin, NIS or EpCAM and stained less intensely with DAPI (Fig. 5 *d*). These differences may represent heterogeneity of expression of biomarkers within the cells or the second group of cells may be undergoing cell death. The diameter of the circulating thyroid cancer cells was 16 ± 0.3 µm.

CTCs were detected in blood from four out of six patients with ovarian cancer. The cells all expressed EpCAM and cytokeratins. CA‐125 expression was detected in around half of the tumour cells (Fig. 5 *e*). The diameter of the CTCs detected in blood from ovarian cancer patients was 13.6 ± 0.59 µm. This diameter was significantly smaller than the diameters of CTCs detected in oesophageal adenocarcinoma, thyroid cancer and hepatocellular carcinoma patients (*p* < 0.001).

## Discussion

We report a method for the detection and accurate characterisation of CTCs by high‐resolution image flow cytometry. We demonstrate that this method is reproducible in samples from four tumour types. EpCAM was included within our panel of antigens, but could be replaced with other bio‐markers for detection of nonepithelial malignant cells. Similarly as novel biomarkers are discovered, analysis of these could be incorporated. The method could be adapted also for measurement of pharmacodynamic biomarkers. The process of enrichment that we describe is based exclusively upon the positive depletion of haematological cells. Following this depletion, CTCs are distinguished from residual leukocytes and cellular debris by analysis of the expression of multiple antigens and by examination of cellular morphology in the high quality images.

The main focus of CTC research has been the value of CTC enumeration for prognosis discrimination in patients with metastatic disease and for prediction of response to cytotoxic therapy. Levels of CTCs are associated with overall survival in pre‐ and on‐treatment patients with metastatic breast cancer, metastatic colorectal cancer and castration‐resistant prostate cancer.^2^, ^3^, ^14^, ^15^, ^16^, ^17^, ^18^, ^19^ The numbers of CTCs detected in patients with metastatic cancer are often low, and because detection of a single CTC may determine whether a patient is categorised into a good or a bad prognostic group,^2^, ^3^ it is important that all CTCs are detected, not only specific subpopulations. A strength of our method is that it permits detection of heterogeneity within a patient's CTC population (Fig. 5 *d*).

There is considerable interest in the analysis of CTCs as a means of studying the biology and behaviour of metastatic cancer. Metastatic disease is frequently difficult to biopsy and treatment is based usually on analysis of the primary tumour. Specific protein expression in metastatic disease may differ from that of the primary tumour.^20^, ^21^ The capability to detect multiple antigens simultaneously enables detailed molecular characterisation of CTCs and may provide an accurate assessment of the biology of the underlying metastatic disease.

It is important that the specificity of CTC detection is high. There is no consensus definition as to what constitutes a CTC partly because of the large number of techniques used for their detection. Variation in the phenotypic criteria by which CTCs are defined results in different CTC counts with varying degrees of clinical significance.^22^ The majority of studies define CTCs based upon positive and negative antigen expression. In some studies, molecular definition is combined with cell morphology. The high‐resolution imaging that we describe allows us to discriminate objects that might otherwise have been included in the enumeration of CTCs. Some studies^11^, ^23^, ^24^, ^25^, ^26^, ^27^, ^28^, ^29^, ^30^, ^31^, ^32^ have reported substantially larger numbers of CTCs than have been identified in the present study or in other reports.^2^, ^3^ It is possible that such high numbers reflect inclusion of noncellular objects and cellular debris.

Analysis of CTCs without enrichment is achievable with an ImageStream^X^ flow cytometer, but the time required to process an unenriched sample precludes realistic routine clinical application. Analysis of one unenriched 5 ml blood sample would take 15 hr compared to 1 hr 40 min for an enriched sample. The losses in our method occur predominantly during the enrichment and antigen detection stages. The recovery of cells during the final analysis with the ImageStream^X^ flow cytometer is 89.2%. The overall loss from the procedure is 44.8%, of which 38.1% is lost during the enrichment and 6.7% during the image collection. The EasySep CD45 depletion kit achieved the optimal recovery of CTCs without generating large quantities of cellular debris. Recovery rates are comparable with other methods in which leukocytes are depleted positively^33^, ^34^ and were consistent over a range of cell concentrations for all tumour types. Leukocyte depletion in head and neck cancers has been evaluated by fluorescent activated cell sorting with a variety of commercially available anti‐CD45 antibodies and magnetic particles including EasySep.^34^ Recovery rates of up to 86% were reported but in these analyses, cells were added to buffy coat rather than whole blood, which should give higher recovery rates because the losses associated with red cell lysis are not considered.^35^

The most widely used method for cell‐based CTC analysis is the CellSearch system (Veridex) which has FDA approval for use in metastatic breast, colorectal and prostate cancer. Enrichment of samples depends upon positive selection of CTCs that express EpCAM, which means that detection of malignant cells is limited to those of epithelial origin that express EpCAM. There is considerable heterogeneity in EpCAM expression in established epithelial cancer cell lines.^36^ Epithelial tumour cells that undergo epithelial mesenchymal transition (EMT) lose EpCAM expression and EpCAM expression changes during the cell cycle.^37^

A single study has attempted comparison of the ImageStream^X^ and CellSearch^38^ by analysis of PANC‐1 pancreatic cancer cells and reported that the accuracy of enumeration was lower with the ImageStream^X^. The authors enriched the PANC‐1 cells with different methods prior to analysis with the two platforms which, because the method of enrichment affects recovery rates, means that the two detection rates are difficult to evaluate.

There are few reports in the literature about the analysis of CTCs in oesophageal adenocarcinoma. Survivin mRNA was detected by RT‐PCR in peripheral blood of patients with a variety of gastrointestinal tumours including oesophageal adenocarcinoma.^39^ A recent study using the CellSearch platform assessed CTC numbers in patients with advanced oesophagogastric adenocarcinoma undergoing palliative chemotherapy.^40^ The study was ended prematurely due to the loss of commercial funding. In 11 patients with advanced oesophageal or oesophagogastric junctional tumours, four were found to have CTCs. In thyroid cancer, there is again a lack of evidence for the value of CTC detection. A single study evaluated the detection carcinoma embryonic antigen (CEA) mRNA by RT‐PCR in 121 patients undergoing surgery for thyroid cancer^41^ and detected CTCs in 5% of patients.

EpCAM‐positive CTCs were detected with the CellSearch system in 18 of 59 patients with hepatocellular carcinoma^42^ and an association between presence of CTCs and overall survival reported. In another study, CTCs were found in 28% of HCC patient samples analysed with the CellSearch system but in 100% of samples analysed with an EpCAM‐independent filtration method.^43^ In a third study, multi‐immunofluorescence identified considerable heterogeneity within CTC populations in HCC patients^44^ and changes in the ratio of epithelial to mesenchymal cells were associated with a longer time to disease progression.

There is more extensive literature about the role of CTCs in ovarian cancer patients. Several studies have isolated a mononuclear cell fraction by density gradient separation followed by positive selection of CTCs based on their expression of epithelial antigens, usually a single antigen.^45^, ^46^ In one study in which cells were enriched sequentially by epithelial and leukocyte‐specific antigen expression, up to 149 CTCs were detected per millilitre of blood in 61% of patients under evaluation for ovarian cancer.^47^ A wide variation in the detection rates of CTCs in patients with ovarian cancer of between 12 and 100% have been reported.^5^, ^46^, ^48^, ^49^ The highest number of CTCs reported is 3,118/ml of blood.^45^

A large number of methods with different strengths are described for the enumeration and characterisation of CTCs. It is unlikely that a single technique will be suitable for all research and clinical applications. The principle strengths of the method we describe are the quality of the images produced, the lack of positive selection of CTCs, its applicability to all tumour types, and the ability to characterise the biology of the cancer cells.
